# Exosomal miR‐27 negatively regulates ROS production and promotes granulosa cells apoptosis by targeting SPRY2 in OHSS

**DOI:** 10.1111/jcmm.16355

**Published:** 2021-02-27

**Authors:** Kailu Liu, Weijie Yang, Mengting Hu, WenXiu Xie, Jingyu Huang, Meiting Cui, Xi He, Xiaowei Nie

**Affiliations:** ^1^ Department of Reproductive Medicine Jiangsu Province Hospital of Chinese Medicine Affiliated Hospital of Nanjing University of Chinese Medicine Nanjing China; ^2^ Assisted Reproduction Unit Key Laboratory of Reproductive Dysfunction Management of Zhejiang Province Department of Obstetrics and Gynecology Sir Run Run Shaw Hospital Zhejiang University School of Medicine Hangzhou China; ^3^ State Key Laboratory of Reproductive Medicine Nanjing Medical University Nanjing China; ^4^ State Key Laboratory of Reproductive Medicine Clinical Center for Reproductive Medicine The First Affiliated Hospital of Nanjing Medical University Nanjing China; ^5^ Department of Human Anatomy and Histoembryology Nanjing University of Chinese Medicine Nanjing China

**Keywords:** exosome, follicular fluid, miRNAs, OHSS

## Abstract

Ovarian hyperstimulation syndrome (OHSS) is one of the most dangerous iatrogenic complications in controlled ovarian hyperstimulation (COH). The exact molecular mechanism that induces OHSS remains unclear. In recent years, accumulating evidence found that exosomal miRNAs participate in many diseases of reproductive system. However, the specific role of miRNAs, particularly the follicular fluid‐derived exosomal miRNAs in OHSS remains controversial. To identify differentially expressed follicular fluid exosomal miRNAs from OHSS and non‐OHSS patients, the analysis based on miRNA‐sequence was conducted. The levels of 291 miRNAs were significantly differed in exosomes from OHSS patients compared with normal control, and exosomal miR‐27 was one of the most significantly down‐regulated miRNAs in the OHSS group. By using MiR‐27 mimic, we found it could increase ROS stress and apoptosis by down‐regulating the expression of p‐ERK/Nrf2 pathway by negatively regulating SPRY2. These data demonstrate that exosomal miRNAs are differentially expressed in follicular fluid between patients with and without OHSS, and follicular fluid exosomal miR‐27 may involve in the pathological process of OHSS development.

## 1 INTRODUCTION

Ovarian hyperstimulation syndrome (OHSS) is a grievous complication of assisted reproductive technologies (ART), which is ordinarily induced by abnormal reaction to human chorionic gonadotropin (hCG).^1^ Statistical data reveals that 32% of the patients in the general IVF cycle suffered mild OHSS, 10%‐15% exhibited moderate OHSS, while 5%‐8% of the patients were diagnosed with severe OHSS.^2^ Besides influencing clinical outcomes like embryo implantation rates and live birth rates,^3^ OHSS could cause life‐threatening conditions, such as acute renal insufficiency, acute respiratory distress, in severe cases, it causes venous thrombosis and mortality.^4^, ^5^ With the development of ovulating induction protocols, women undergoing GnRH antagonist stimulation protocols partially decreases the risk of OHSS, but some deficiencies still limit the application of this method.^6^ So far, prevention strategies and the development of novel therapeutic targets for OHSS remains a challenge in reproductive medicine.

The exosome is a small vesicle with lipid bilayer structure secreted by cells with a diameter of about 50‐150 nm, it carries DNA, mRNA, microRNA, and proteins.^7^ Studies have shown that exosomes can be detected in various body fluids, such as blood, urine,^8^ cerebrospinal fluid,^9^ ascites, follicular fluid,^10^ amniotic fluid and joint fluid.^11^ Animal experiments have proved that the microRNAs in follicular fluid of mature follicles are distinct from those in immature follicles. When cultured in follicular fluid, these microRNAs could be absorbed by follicular cells.^12^ Recent studies have reported that exosomal microRNAs derived from follicular fluid are directly linked to granulosa cells apoptosis, follicular development, and ovarian function.^13^, ^14^ Also, Wang and his colleagues sequenced exosomal circRNAs in PCOS follicle fluids, they identified 167 up‐regulated, 245 down‐regulated circRNAs and constructed a circRNA‐microRNA network.^15^ However, there was no evidence on the expression profile of exosomal microRNAs in follicular fluid and their potential roles in OHSS development.

In this work, we identified differentially expressed exosomal miRNAs (follicular fluid derived) from women with or without OHSS. We found that miRNA‐27‐3p was the most significantly reduced in OHSS group, and interacted with SPRY2 in granulosa cells. Further, we found that miR‐27 inhibits Nrf2 expression via EGFR/ERK signal and aggravates cells apoptosis by increasing ROS production. Our study shows a possible mechanism of exosomal miR‐27‐3p on the microenvironment of the follicle, which probably involved in the OHSS development.

## 2 MATERIALS AND METHODS

### 2.1 Characteristics of participants

All infertile women were treated with IVF‐ET from June 2017 to November 2017 in the Reproductive Medical Center of The First Affiliated Hospital of Nanjing Medical University. Our experimental scheme was approved by the Ethics Committee of the First Affiliated Hospital of Nanjing Medical University (Ethics No. 81370764). All the research participants signed the informed consent of the patients. Patients who participated in the study received long‐term regimen therapy. The diagnostic criteria of OHSS were borrowed from Golan criteria.^16^ For the OHSS patients, the inclusion criteria were as follows: serum AMH > 5 ng/mL, serum E2 > 3500 pg/mL on HCG day and oocytes > 20. The controls were diagnosed with fallopian tubal diseases via laparoscopy and hysteroscopy, or exhibited male factor infertility. Women suffering from malignancy, benign ovarian cyst including endometrioma, allergic diseases; pelvic inflammation, known chronic, systemic, metabolic, or endocrine disease excluding polycystic ovarian syndrome, were excluded from this study.

### 2.2 Collection of follicular fluid

All patients received 10 000 IU human chorionic gonadotropin (hCG) administration on trigger day. The follicular fluid from the large follicle (>18 mm) of the patient first sampled and collected in a 15 mL centrifugal tube for centrifugation (Falcon). After 2500 g centrifugation for 15 minutes, we collected the supernatant and stored at –80°C until use. Granulosa cells were purified using density centrifugation from follicular fluid at 2000 g for 10 minutes. The cell pellets were re‐suspended in percoll (GE Healthcare) and centrifuged at 2000 g for 20 minutes to separate red blood cells. The interface cells were collected for future use.

### 2.3 Exosome isolation and identification

Follicular fluid exosomes were isolated and characterized according to a previously published protocol.^17^ The follicular fluid was gradually melted on ice and diluted by adding 20 mL PBS (pH 7.4) (Thermo Fisher Scientific). The follicular fluid was isolated by centrifugation at 2500 g for 30 minutes, and the supernatant was then transferred to a new centrifugal tube and centrifuged at 12 000 g for 5 minutes to eliminate large particles. After centrifugation, we filtered the supernatant using a 0.22 μm filter. Finally, the filtered samples were transferred to ultracentrifuge tubes for centrifugation at 120 000 g and 4°C for 4 hours in an ultracentrifuge (Beckman Coulter). Thereafter, exosome pellets were re‐suspended in RIPAlysate (Thermo Fisher Scientific) for western blot analysis or in PBS for nanoparticle tracking analysis (NTA). Then, the exosomes were collected for further treatment. Through the dynamic light scattering method, the particle size distribution of exosomes was evaluated. We entrusted the NTA analysis to Shanghai XP Biomed Ltd.

### 2.4 Exosomal RNA extraction and sequencing

The exosomes were added into 1 mL Trizol (Invitrogen, USA), blown uniformly with RNA‐free gun‐head repeatedly, and then placed at room temperature for 5 minutes. A total of 200 μL chloroform was added and incubated at room temperature for 10 minutes with severe shock for 15 seconds. Then, the exosomes were centrifuged at 12 000 g and 4°C for 15 minutes and 400 μL supernatant collected. Additional 400 μL isopropanol (Sangon Biotech) was added, mixed well, and left to dry at room temperature for 10 minutes. Afterwards, the mixture was centrifuged at 12 000 g and 4°C for 10 minutes and the supernatant discarded. Again, 1 mL 75% ethanol was added, gently washed and precipitated, centrifuged it at 4°C for 5 minutes and the supernatant discarded. After drying at room temperature, an appropriate amount of DEPC water was added to dissolve and kept on ice for the next experiments.

Follicular fluid samples from ten OHSS patients and ten normal controls were used for exosomal RNA sequencing. Library preparation and sequencing were conducted at Anoroad. A total of 1 μg total RNA per sample was used for the small RNA library. After the total RNA samples were quantified, the RNA fragments were fractionated on a 15% polyacrylamide gel (Invitrogen) and small RNAs ranging between 15 and 30 nucleotides (nt) were used for library preparation. The two ends of the separated RNA fragments were connected respectively. Small RNAs were reverse transcribed by RT primers and amplified by PCR. The PCR products were sequenced using the Illumina HiSeq 2500 platform (Illumina Inc).

### 2.5 Detection of microRNAs

The samples were isolated and reversed by reverse transcription AMV enzyme reverse transcription system (Taraka). The 10 μL of a master mix contains 2 μL of AMV 5X buffer, 0.5 μL AMV Enzyme, 1 μL dNTPs, 1 μL reverse transcription primer, 1 μL RNA template, and 3.5 μL DEPC Water. The samples were incubated at 16°C for 30 minutes followed by 42°C for 30 minutes and 85°C for 15 minutes. The resulting cDNA was collected for real‐time PCR. Real‐time PCR was performed using 10 μL SYBR Premix ExTaq (2X), 0.8 μL forward primers (5 pmol/mL), 0.8 μL reverse primers (5 pmol/mL), 0.4 μL ROX, 1 μL reverse transcription reaction products, and 7 μL ddH_2_O. The following two‐step PCR amplification was used: 95°C for 5 seconds and 60°C for 30 seconds for a total of 40 cycles. After the reaction, the amplification and melting curves of Real‐time PCR were confirmed, and the data were analysed by the 2^−ΔΔCT^ method.

### 2.6 MicroRNAs target gene ontology and pathway analysis

Differential expression of known and unknown microRNAs was analysed and target genes of differentially expressed microRNAs were predicted on the miRNA‐database. Further, GO annotation analysis and KEGG signalling pathway enrichment analysis was performed to establish the enriched GO term and Pathway. Eventually, the interaction of microRNAs with genes in the GO term and Pathway was analysed, and the key pathways and regulatory networks in OHSS were mapped by combining the known signalling pathways. The regulatory relationship between the two was obtained on the microRNA database, and the microRNA‐RNA interaction network was established.

### 2.7 Cell culture and transfection

A steroidogenic human granulosa‐like tumour cell line (KGN) was used, the cells were cultured in six‐well plates with DMEM/F12 media (Thermo) containing 1% penicillin/streptomycin, 10% foetal bovine serum (complete medium), 100 mg/mL streptomycin sulphate (Thermo), and 1X GlutaMAS (Thermo). The MiR‐27‐3p mimics, miR‐27‐3p inhibitor, and miR‐27‐3p NC were designed and synthesized by Ribobio. MiR‐27‐3p mimics were then transfected at 50 nmol/L and 100 nmol/L, miR‐27‐3p inhibitors were transfected at 100 nmol/L, using transfection Reagent (Giagen). KGN cells were treated with 100 and 50 μg/mL exosomes or PBS as a control. The KGN cells were collected for RNA or protein studies. After 48 hours of incubation, we obtained the transfected cells. The experiments were repeated independently in triplicate.

### 2.8 Small interfering RNA (siRNA) transfection

Cells were transfected with 100 nmol/L SPRY2 siRNA (sc‐41037, Santa Cruz) or control siRNA (sc‐37007, Santa Cruz) using Lipofectamine 2000 according to the manufacturer's instruction.

### 2.9 Cell proliferation assay

Cell proliferation Assay was tested using the CCK8 Assay. All processes were according to the manufacture's protocol (Beyotime). The assessment of cell viability was performed by BeyoClick EdU Cell Proliferation Kit with Alexa Fluor 488 (Beyotime). 100 μmol/L EdU solution was added to the culture medium, and cells were cultured for 2 hours. Hoechst 33342 was used for nuclear staining according to the manufacturer's instructions. Cells showing both green and blue stains were identified as EdU‐positive cells. All cells were examined by flow cytometry.

### 2.10 Assessment of apoptosis

Granulosa cells were transfected for 48 hours, stained with Tunel, and morphologically assessed using a fluorescent microscope. At least 200 cells in a selected area were counted in each treatment group, with individuals counting blinded to identity. Normal cells show round and intact nuclei whereas apoptotic cells exhibit morphological changes with karyopyknosis or fragmentation.

### 2.11 RNA extraction and qRT‐PCR

Total RNA was extracted from the granulosa cells and 293T cells using Trizol following the manufacturer's protocol and quantified using the NanoDrop ND‐2000 spectrophotometer. Total RNA was reverse transcribed into cDNA using RNA reverse transcription kit (Thermo). For the detection of genes, qPCR was performed as previously described. The primers for genes are listed in Table 1.

**TABLE 1:** PCR primer

Gene	NCBI GeneID	Primer sequence
human‐VEGF	7424	F: AGGGCAGAATCATCACGAAGT R: AGGGTCTCGATTGGATGGCA
human‐SPRY2	10253	F: ATGGCATAATCCGGGTGCAA R: TGTCGCAGATCCAGTCTGATG
human‐SOX5	6660	F:ATAAAGCGTCCAATGAATGCCT R: GCGAGATCCCAATATCTTGCTG
human‐SATB1	6304	F: CCAGGTTGGAAAGTGGAATCC R: GGGGCAACTGTGTAACTGAAT
human‐RECK	8434	F: TGTGAACTGGCTATTGCCTTG R: GCATAACTGCAACAAACCGAG

### 2.12 Dual‐luciferase reporter assay

The relationship between the SPRY2 and miR‐27‐3p was verified on the database Tarcan. Next, the luciferase reporter plasmid containing wild‐type 3’‐UTR of SPRY2 was purchased from Genomeditech. Wild‐type and the mutated vectors were co‐transfected with miR‐27‐3p mimics or negative control into HEK‐293T cells using Lipofectamine2000. After 48 hours, Luciferase activity of cultured supernatant was measured using a kit as per the manufacturer's instructions.

### 2.13 Immunofluorescence

Granulosa cells were collected and washed with PBS three times at room temperature. They were then placed in PBS containing 0.1% Triton X‐100 at room temperature for 30 minutes. After blocking in PBS‐5% bovine serum albumin (BSA) at 37°C for 1 hour, the granulosa cells were incubated with the special antibodies described above at 4°C overnight. Cells were further incubated with goat anti‐mouse and goat anti‐rabbit at 37°C in the dark followed by staining of nuclei with DAPI for 5 minutes. Finally, all granulosa cells were placed on slides with mounting medium (Beyotime) and gently covered with ethanol‐primed coverslips and kept in the dark until confocal scanning.

### 2.14 Co‐Immunoprecipitation

The cells were washed with PBS at room temperature and collected by centrifugation. The PBS was drained carefully and 100 μL non‐denaturing lysis buffer added. Further, we added a 10 μL antibody and topped with Lysis Buffer containing protease inhibitors to make 500 µL volume. The mixture was gently mixed overnight at 4°C on a rotary mixer. The Protein‐A/G beads were washed twice with 1 mL Wash Buffer and centrifuged at 2000 g for 2 minutes aspirating the supernatant in between washes. The sample was suspended as a 50% slurry in Wash Buffer. After Antibody Binding, 40 µL of protein‐A/G beads slurry was added to each tube and incubated at 4°C for 1 hour. The beads were collected by low‐speed centrifugation at 2000 g and 4°C for 2 minutes. Then, the beads were washed 3 times with 1 mL Wash Buffer and collected by low‐speed centrifugation at 4°C aspirating the supernatant in between washes. Afterwards, the Wash Buffer was removed and the beads kept wet. A total of 40 µL 2x SDS‐PAGE loading buffer was added to the beads and boiled for 5 minutes to elute the complex. Eluent collected after centrifugation was stored on ice for same‐day analysis or frozen at −80˚C for future analysis by SDS‐PAGE.

### 2.15 Western blot analysis

The samples (50 μg protein) were lysed in SDS sample buffer with 1 mmol/L PMSF and separated in a 12% SDS‐PAGE gel in running buffer at 200V for 30 minutes. Protein standards were included (Thermo Fisher Scientific). Proteins were electro‐transferred onto PVDF membranes (Millipore), and the membranes were blocked with blocking buffer (Beyotime) for 30 minutes. Primary anti‐CD63 (Abcam, 1:500 dilution), anti‐Alix (Abcam, 1:1000 dilution), anti‐TSG101(Abcam, 1:500 dilution), anti‐CD9 (Abcam, 1:200 dilution,), anti‐βactin (Abcam 1:2000 dilution), anti‐GM130 (Abcam), 1:500 dilution, anti‐SPRY2 (Proteintech, 1:200 dilution), anti‐EGFR (Proteintech, 1:500 dilution), anti‐VEGF (Proteintech, 1:500 dilution), anti‐Bcl‐2 (Proteintech, 1:1000 dilution),anti‐Bax (Proteintech, 1:1000 dilution) and anti‐active caspase3 (Proteintech, 1:100 dilution), anti‐ERK (CST, 1:500 dilution), anti‐p‐ERK (CST, 1:500 dilution), anti‐Nrf2 (Proteintech, 1:100 dilution), Keap1 (Proteintech, 1:500 dilution) were used. The primary antibody was incubated for 2 hours at 4°C. Membranes were then washed 3 times in TBST for 10 minutes after secondary antibodies incubation step, and the bands detected by enhanced chemiluminescence (Thermo Fisher Scientific).

### 2.16 Caspase 3/7 activity analysis

To detect the activity of caspase‐3/7 in KGN cells, we used the Caspase3/7 activity apoptosis assay kit (Ribobio) according to the manufacturer's instructions. We added different groups of exosomes in the KGN cells culture for 48 hours. We incubated the cells with caspase 3/7 assay solution for 45 minutes, and then analysed with a flow cytometer.

### 2.17 ROS staining analysis

ROS analysis was conducted using H2DCFDA (Sigma, USA). Granulosa cells were incubated in the dark with 5uM staining solution in PBS at 37°C for 30 minutes and harvested with a 0.05% trypsin‐EDTA solution, then immediately analysed with a flow cytometer.^18^

### 2.18 Statistical analysis

For data analysis, we used SPSS 19.0 statistical software. Chi‐square test was used for counting data, while one‐way ANOVA for comparison between groups. The measurement data were expressed in the form of mean ± SD. The comparison between the two groups was analysed by the Student's t‐test. The difference at *P* <0.05 was statistically significant.

## 3 RESULTS

### 3.1 Participant characteristics

The characteristics of all the enrolled participants are summarized in Table 2. As tabulated, there is no significant difference between OHSS patients and normal controls in their age, basal FSH, stimulation days of gonadotropin, and duration of infertility. The OHSS patients showed a significant up‐regulation of the values of basal LH, basal LH/FSH, estradiol on the hCG day, number of oocytes and AMH level (*P* <0.05).

**TABLE 2:** Comparison of clinical features between the two groups

	OHSS	Normal Control	*P*
Number	15	15	
Age(years)	29.07 ± 3.85	31.20 ± 4.28	0.16
Duration of infertility(years)	1.66 ± 0.72	1.73 ± 0.70	0.80
Number of oocytes	13.00 ± 3.98	5.26 ± 2.19	<0.001
Basal FSH(U/L)	5.37 ± 2.35	6.91 ± 1.76	0.05
Basal LH(U/L)	5.26 ± 2.57	3.48 ± 1.64	<0.05
AMH(ng/mL)	12.12 ± 4.32	3.55 ± 0.86	<0.001
Oestradiol on day of HCG(pg/mL)	13 319 ± 400	2840.25 ± 20	<0.01
Dose of gonadotropins	1577 ± 633.8	2192 ± 609.6	<0.05
No. of stimulation days	10.73 ± 1.83	10.8 ± 1.32	0.90

Note

*P*: There was no statistical difference in each index between groups.

### 3.2 Characteristics of follicular fluid exosomes

**FIGURE 1:**
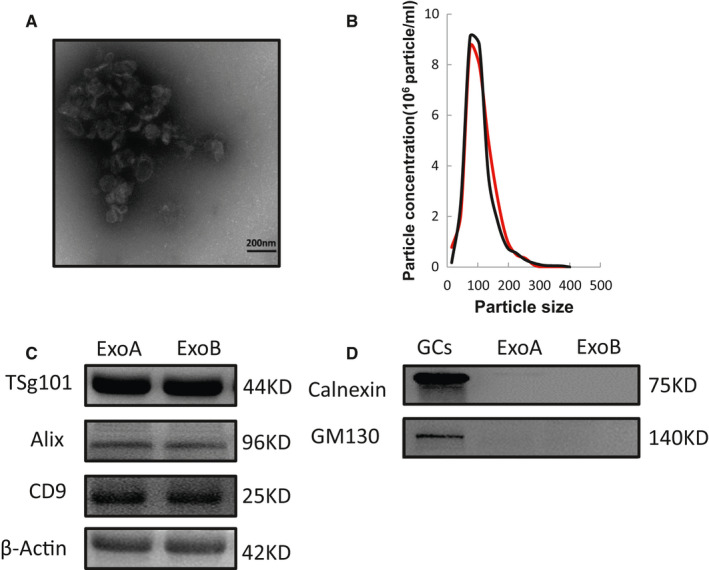
Characterization of exosomes isolated from the human follicular fluid. A, Follicular fluid‐derived exosomes were examined by negative staining on transmission electron microscope. (scale bar, 200 nm). B, Nanoparticle tracking analysis profile of exosomes indicated the distribution of diameter size. The red line and black line represent OHSS group and normal control group. (C,D) Western blots of positive exosomal markers of exosomes (Alix, Tsg101, and CD 9) and two negative marker of exosomes (Calnexin and GM130). ExoA:OHSS group; ExoB: normal control group. GCs: human granulosa cells

Exosomes have a typical round or elliptical cup‐disc structure with a diameter of 30‐100 nm (Figure 1A). We further characterized the follicular exosomes size and the purity by using NanoSight analysis (NTA). NTA of exosome size showed that the diameter of exosomes in the follicular fluid of the two groups ranged between 75 to 150 nm. Two colours were used two to distinguish them. The red line represents the OHSS group while the black line represents the normal controls (Figure 1B). A total of 3 positive marker proteins (Alix, CD9, Tsg101) and two negative marker proteins (Calnexin, GM130) were selected to validate the follicular fluid exosomes of the two groups by western blot. Alix, CD9, and Tsg101 were all expressed in the exosome samples (Figure 1C), but exosome lysates showed no expression of Calnexin and GM130 (Figure 1D). These results attested that the purified particles shared the typical features of exosomes.

### 3.3 Follicular fluid exosomes from non‐OHSS group inhibit cell viability and increase apoptosis in human KGN cells

**FIGURE 2:**
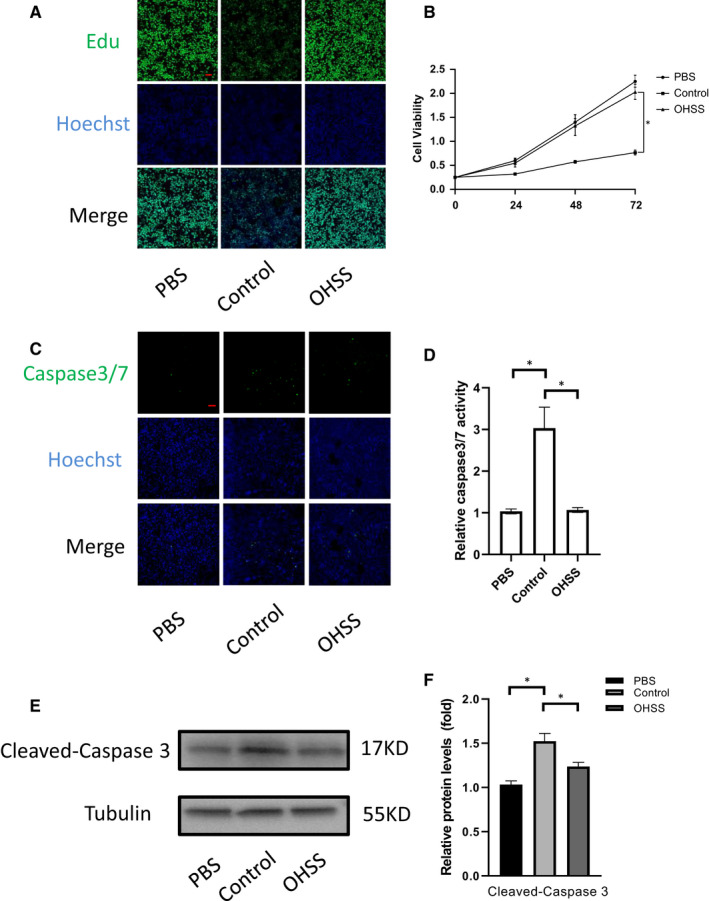
Normal control group exosomes inhibits cell viability and promotes apoptosis in KGN cells. (A,B) Edu staining analysis of KGN cells. KGN cells were transfected with OHSS group and normal control group exosomes for 24, 48 and 72 hours. (scale bar, 50 μm). (C,D) KGN cells were transfected with exosomes for 48 h, and apoptotic cells were measured by Caspase3/7 activity kit. Relative caspase3/7 activity was calculated by average positive cell ratio (positive cells / total cells) and standardized the data with PBS group as the internal control. The results were shown as means ± SD (n = 3). (scale bar, 50 μm). (E,F) Western blot analysis of cleaved caspase 3 protein level(normalized to TUBULIN) after transfection with two groups exosomes for 48 h. The results were shown as means ± SD of three independent experiments. *<0.05.

To explore the effects of exosomes on KGN cells viability, we transfected cells with exosomes from the two groups for 48 hours. We used Edu staining to examine the effects of exosomes on the viability of KGN cells. We witnessed that, treatment with normal control group exosomes markedly decreased granulosa cell viability at both 48 and 72 hours. In contrast, treatment with OHSS group exosomes did not influence cell viability (Figure 2A,B). Next, we assessed the effects of treatment with exosomes on KGN cells apoptosis by using Caspase3/7 activity apoptosis kit and Western blot. It was found that the transfection of KGN cells with normal control group exosomes increased the percentage of apoptotic cells (Figure 2C‐F). Conclusively, these findings suggest that exosomes from the normal control group inhibit viability and enhance apoptosis of KGN cells.

### 3.4 Differential expression of miRNAs in follicular fluid exosomes derived from OHSS and non‐OHSS patients

**FIGURE 3:**
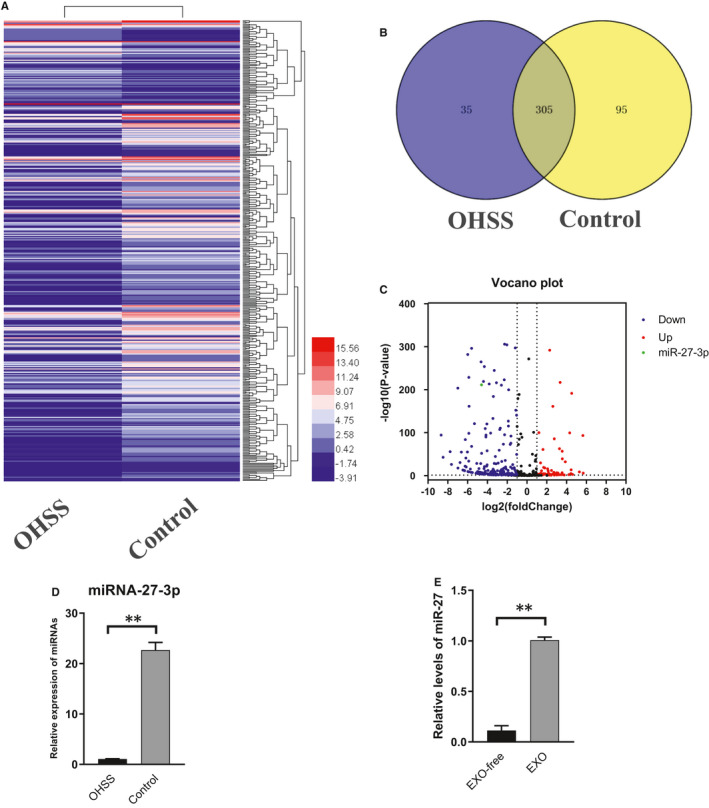
The expression of miRNA‐27 in two groups of exosomes. A, Heat map of differentially expressed exosomal miRNAs from OHSS and normal control group. B, Venn diagrams of differentially expressed miRNAs. C, The Volcano map showing the distribution of differential miRNAs between the OHSS group and normal control group according to their *P* values and fold‐changes. Candidates with *P* <0.05 and |log 2(fold‐change)| ≥1 are considered differential. Red point indicated the 78 up‐regulated miRNSs, blue point indicated the 213 down‐regulated miRNAs in OHSS group. Black point represented the miRNAs that were no difference between OHSS and normal control samples, green point represented miR‐27. D, Exosomal miR‐27‐3p expression in OHSS and normal control groups of exosomes. (1.18 ± 0.24 vs 21.06 ± 2.60) (Mean ± SD, **<0.01,t‐test, n = 3). E, Relative expression of miR‐27 in exo‐free follicular fluid and exosomes. (0.11 ± 0.04 vs 1.007 ± 0.03) (Measn ± SD, **<0.01, t‐test, n = 3)

To identify differentially expressed miRNAs in exosomes between two groups, we isolated total RNA from two groups of follicular fluid exosomes and constructed a small RNA library. OHSS group follicular fluid exosome sequencing profiling identified 526 miRNAs, while we found 626 miRNAs in the normal control group, all of which satisfied *P* <0.01 as shown in the heatmap (Figure 3A). The total number of co‐expression miRNAs was 305 (Figure 3B). And between two groups, there were 291 differently expressed miRNAs among them, 78 up‐regulated miRNAs and 213 down‐regulated miRNAs (fold‐change ≥ 2, *P* value < 0.05) (Figure 3C). To understand the role of miRNAs in OHSS group, functional enrichment analysis was applied in target genes of differentially expressed miRNAs in patients with OHSS compared to normal controls. The target genes of microRNAs were predicted using the microRNA and a (3.3a) database, and we explored the GO (Gene Ontology) analysis and enriched KEGG pathways of the predictive target genes. The first 5 items of up‐regulation and down‐regulation are listed respectively (Figure S1A,B). Of the differently expressed miRNAs, miR‐27‐3p was significantly down‐regulated (Figure 3D), and was significantly higher in exosomes compared with follicular fluid supernatant in normal control (Figure 3E).

### 3.5 SPRY2 is a potential direct target of miR‐27‐3p

**FIGURE 4:**
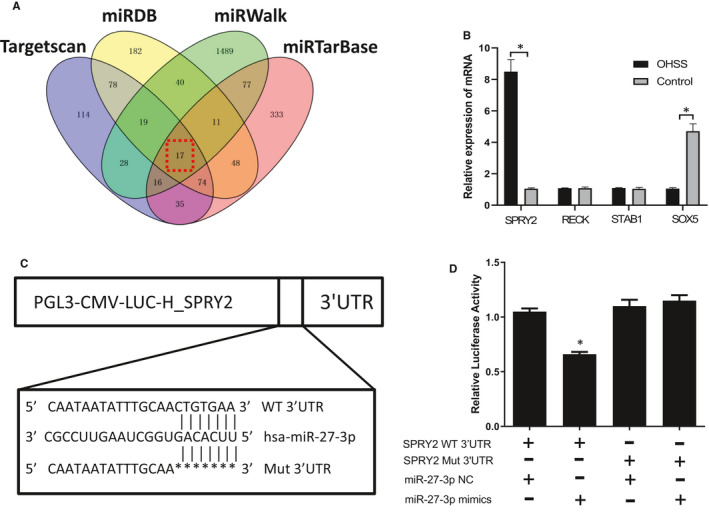
Exosomal miRNA‐27‐3p could target SPRY2. A, Venn figure of the number of predicted target genes of miR‐27‐3p from miRNA target prediction software (TargetScan, miRDB, miRWalk and miRTarBase). Red dotted box represented the common predicted targets. B, The four predicted targets were verified by human granulosa cells mRNA in two groups. (Mean ± SD, *<0.01,t‐test, n = 3). C, D, Targeting of SPRY2 by miR‐27‐3p was confirmed by a dual‐luciferase reporter assay. Luciferase activity in each group of 293T cells. Compared with the miR‐27‐3p‐NC + SPRY2‐3’UTR, miR‐27‐3p‐NC + SPRY2‐3’UTR Mut and miR‐27‐3p mimics + SPRY2‐3’UTR Mut groups. Mean ± SD, Student's t‐test. * <0.05, n = 3

After searching at the online target gene databases (TargetScan, miRanda, miRWalk, and miRTarBase) (Figure 4A), we downloaded 4 potential targets which have the highest comprehensive score after analysing the predicted results (SPRY2, RECK, STAB1 and SOX5). We compared the mRNA expression levels of these 4 genes in patients’ granulosa cells between the two groups, and SPRY2 is speculated to be a target gene of miR‐27‐3p (8.49 ± 0.75 vs 1.05 ± 0.050, *P* <0.05) (Figure 4B). Furthermore, to demonstrate a direct interaction between the SPRY2 3’UTR and miR‐27‐3p, the WT SPRY2 3’UTR region (predicted to interact with miR‐27‐3p) was cloned into a luciferase reporter vector. We also cloned a SPRY2 3’UTR fragment with a mutation of the complementary base (Figure 4C). As expected, luciferase activity suppression was significantly rescinded when interaction between miR‐27‐3p and its target 3’UTR was disrupted in cells transfected with SPRY2 3’UTR Mut. Luciferase activity was reduced by 40% in cells co‐transfected with miR‐27‐3p (Figure 4D).

### 3.6 SPRY2 interacts with EGFR in KGN cells

**FIGURE 5:**
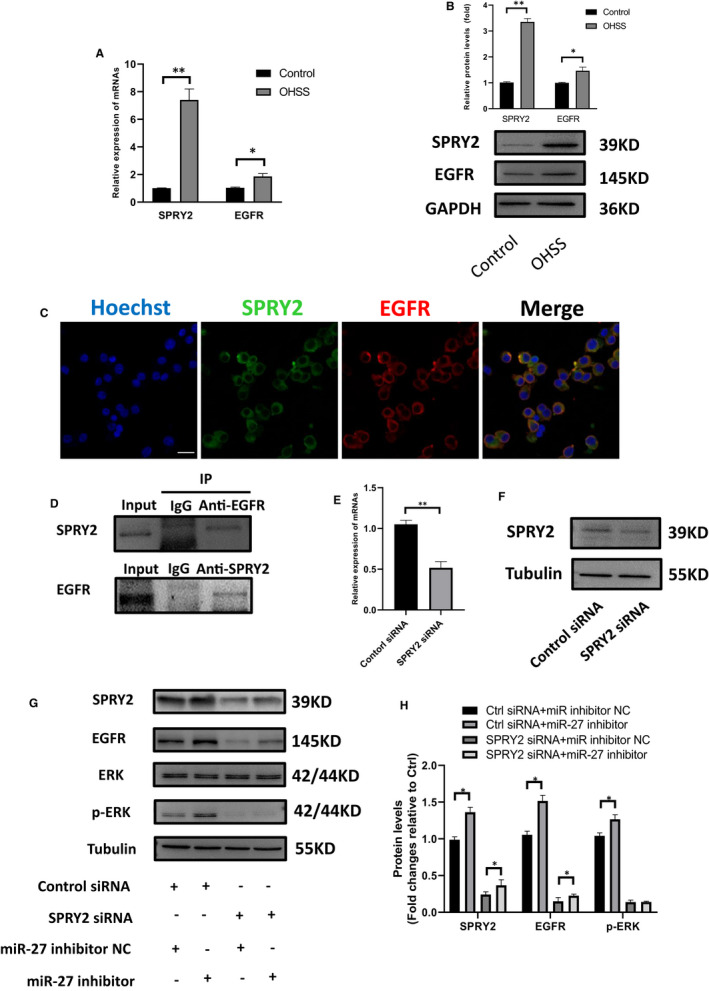
SPRY2 interacts with EGFR in granulosa cells. A, The mRNA expression of SPRY2 and EGFR in two groups of granulosa cells. SPRY2:1.01 ± 0.05 vs 7.4 ± 0.80; EGFR:0.99 ± 0.03 vs 1.02 ± 0.05. (normal control vs OHSS, *<0.05, ** <0.01, n = 3). B, Western blot of SPRY2 and EGFR expression in two groups of granulosa cells. SPRY2:1.01 ± 0.04 vs 3.35 ± 0.13; EGFR:0.99 ± 0.03 vs 1.46 ± 0.14. (normal control vs OHSS * <0.05, ** <0.01, n = 3). C, The distribution of SPRY2 and EGFR in granulosa cells was showed by immunofluorescence. (scale bar, 20 μm). D, Immunocoprecipitation showed that there was interaction between SPRY2 and EGFR. (E,F) The mRNA and protein expression of SPRY2 in KGN cells after transfected with si‐SPRY. ** <0.01, n = 3. (G,H) The protein expression of SPRY2, EGFR, ERK, p‐ERK in the KGN cells after the expression of SPRY2 was knock down. * <0.05, n = 3

SPRY2 belongs to Sprouty protein family and regulates the signalling downstream of multiple growth factor receptors. A search at the STRING database (string‐db.org) for possible interaction proteins of SPRY2 found that EGFR, FGFR2, and GRB2 might interact with SPRY2 (Figure S1C). EGFR was a key receptor in OHSS.^19^ Despite a few literature reporting the interaction between SPRY2 and EGFR in some cells,^20^ it is not clear whether SPRY2 and EGFR interact in granulosa cells. We detected the expression of SPRY2 and EGFR in granulosa cells of two groups by RT‐QPCR and western blot and found that the expression levels of SPRY2 and EGFR in mRNA and protein among the OHSS group was higher than that in the normal control group (Figure 5A,B). Immunofluorescence, co‐localization, and immunoprecipitation results reported an interaction in granulosa cells (Figure 5C,D). According to the previous literature, degradation of EGFR is reduced by SPRY2 binding. Therefore, we knocked down SPRY2 in human KGN cells to verify its role in regulating EGFR function (Figure 5E,F). We examined the expression of EGFR and p‐ERK after knocking down SPRY2 and treating with or without miR‐27 inhibitor. Treatment with miR‐27 inhibitor elevated the expression of EGFR and p‐ERK, and these effects were terminated by pre‐treatment with SPRY2 siRNA. These findings suggest that miR‐27 inhibits KGN cells EGFR and p‐ERK expression by suppressing SPRY2 (Figure 5G,H).

### 3.7 Exosomal miR‐27‐3p promotes KGN cells ROS and apoptosis

**FIGURE 6:**
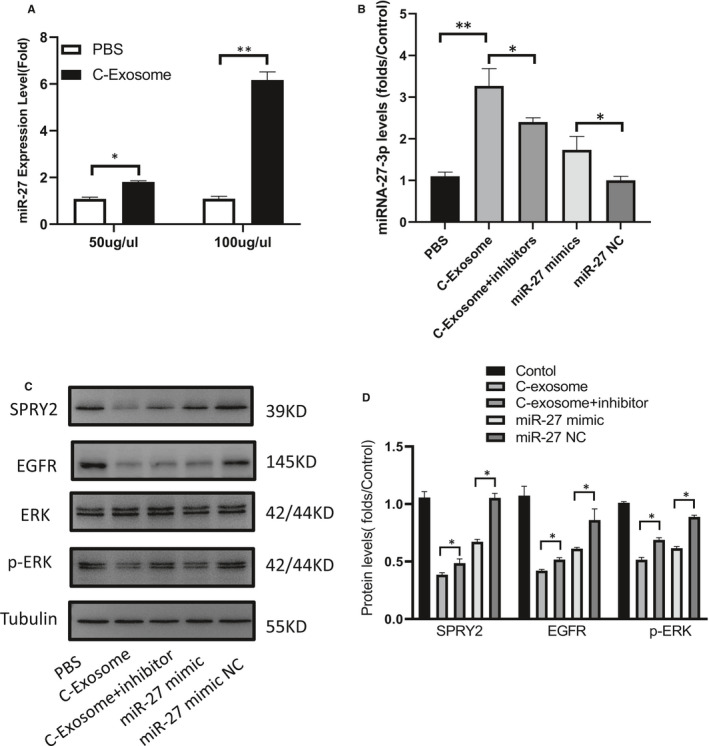
Exosomal miR‐27‐3p inhibited expression SPRY2 in KGN cells. A, The expression level of MiR‐27 in granulosa cells was increased by adding different concentrations of exosomes. * <0.05, ** <0.01, n = 3. B, Effect of normal control group exosome, miR‐27 mimic, miR‐27 inhibitor and miR‐27 NC on KGN cell miR‐27 expression. * <0.05, ** <0.01, n = 3. (C, D) The protein expression of SPRY2, EGFR, ERK and p‐ERK in KGN cells after transfection with normal control group exosome, miR‐27 mimic, miR‐27 inhibitor. * <0.05

**FIGURE 7:**
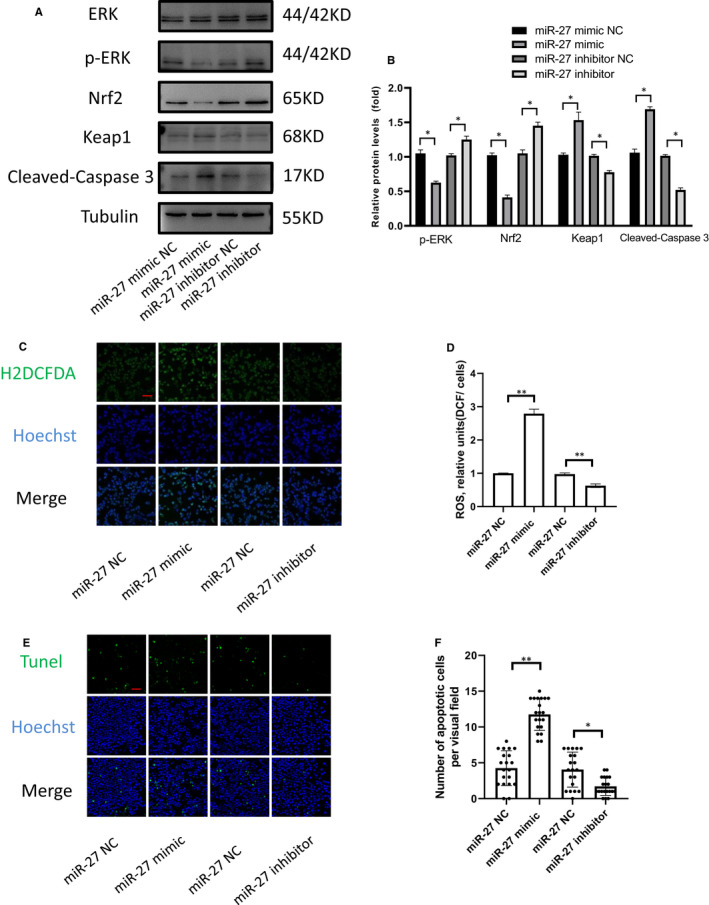
miR‐27‐3p regulates KGN cells Nrf2 expression and promotes KGN cells apoptosis. (A,B) Protein levels of ERK, p‐ERK, Nrf2, Keap1 and cleaved caspase 3 in KGN cells after miR‐27 mimic or inhibitor transfection for 48 h. (C,D) Effect of miR‐27 mimic and mimic‐27 inhibitor on ROS release in KGN cells. Fluorescence intensity of H2DCFDA (green colour) was determined by Image J software. Each value in the graph represents the relative units(fluorescence/cell numbers). (mean ± SD, ** < 0.01, n = 3). (scale bar, 50 μm). (E,F) Tunel staining and number of Tunel‐positive cell in each group. *<0.05, ** < 0.01, n = 3. (scale bar, 50 μm)

The effects of different concentrations of exosomes on the expression level of MiR‐27 in KGN cells were tested and discovered that when the concentration was 100 μg/mL the concentration of MiR‐27 in KGN cells was significantly increased (Figure 6A). In addition, we found that MiR‐27 mimic partly simulate the function of exosomes (Figure 6B). The protein expression of SPRY2 and EGFR were negatively regulated by MiR‐27. Adding MiR‐27 inhibitor to the exosomes of the low‐risk group partially inhibits the effect of MiR‐27 on SPRY2 and the downstream protein EGFR and p‐ERK (Figure 6C,D). EGFR activation could elevate the levels of phosphorylated ERK 1/2. ERK pathway activation promotes cell proliferation and inhibits apoptosis. To explore the role of miR‐27 in mediating granulosa cells ROS and apoptosis, we used miR‐27 mimic and miR‐27 inhibitor. Western blot analysis showed that ERK1/2 phosphorylation and Nrf2 were significantly down‐regulated after transfection with miR‐27 mimic (Figure 7A,B). Under physiological conditions, Nrf2 is repressed by KEAP1. The miR‐27 inhibitor up‐regulated the total Nrf2 expression and down‐regulated the total Keap1 levels in granulosa cells. Through H2DCFDA staining, we detected the ROS levels in granulosa cells. When granulosa cells were transfected with miR‐27 mimic, they showed high oxidative stress (Figure 7C,D). High levels of ROS apoptosis are known to cause apoptosis.^21^ By Tunel apoptosis assay and cleaved caspase 3 expression, we found that miR‐27 increases granulosa cells apoptosis (Figure 7E,F).

## 4 DISCUSSION

Ovarian hyperstimulation syndrome (OHSS) is an iatrogenic condition culminating from the abnormal ovarian response, and it is also closely linked with increased miscarriages and decreased live birth.^22^ Interestingly, OHSS is a good indicator of ovarian function in a sense, since most patients with OHSS are younger than 35 years.^23^ Our statistics also show that OHSS group patients have a higher AMH level, which could reflect the state of ovarian function.^24^ Previously, many studies indicated that the principal reason for OHSS occurrence is that human chorionic gonadotropin (hCG) stimulates ovarian granulosa cells to release a variety of inflammatory factors, such as VEGF, prostaglandin, IL‐6, IL‐8, renin‐angiotensin and prolactin.^25^ In recent times, Li et al reported higher levels of melatonin in OHSS patients follicle fluid compared with normal patients, which suggested that the oxidative stress level of granulosa cells in OHSS patients might be lower than the normal patients.^26^ Granulosa cells are particularly sensitive to ROS, physiological levels of ROS regulate normal function, but excessive oxidative stress in the ovary might lead to abnormal oocyte development^27^ and premature ovarian failure.^28^ In addition, inflammatory factors could induce ROS production. The interactions of increased ROS production and PCOS was observed in previous studies.^29^ Therefore, this raises several timely issues relating to the existence of substances regulating the oxidative stress of granulosa cells in the follicular fluid of OHSS patients and how it affects OHSS development with largely understudied potential.

Exosome‐mediated microRNAs transfer is a major avenue through which genetic material is exchanged between cells.^30^ With the development of technology to extract follicular fluid exosomes, many scholars commenced to study the roles of exosomes in female reproduction.^31^ A study by Hung *et al* found extracellular vesicles from different‐sized follicles differentially stimulate granulosa cell proliferation and mediated PI3K/Akt signalling pathways in granulosa cells.^12^ In contrast to the healthy group, the expression levels of miRNA‐132 and miRNA‐320 in the follicular fluid of PCOS patients were significantly lower.^32^ Recently, the differential long non‐coding RNA expression profiles were reported, researchers discovered that 482 lncRNAs were differentially expressed in granulosa cells of OHSS patients.^33^ Previous study identified miRNAs in exosomes derived from human follicular fluid play important roles in steroidogenesis and closely associated with PCOS.^34^ However, research on the identification and functional characterization of miRNAs in OHSS is still limited.

In this study, we first extracted follicular fluid exosomal miRNAs from patients with or without OHSS and added them into the culture medium to evaluate the effect on proliferation and apoptosis of KGN cells. We established that normal group exosomes inhibit granulosa cell proliferation and promote apoptosis. At present, there are few studies on the function of exosomes extracted from follicular fluid, nonetheless, it has been reported that exosomes from follicular fluid promotes apoptosis of granulosa cells via NF‐kB pathway.^35^ MiRNA‐sequence analysis found 291 miRNAs were differentially expressed between OHSS and normal control. Furthermore, 8 differently expressed miRNAs were identified through RT‐QCR. For further investigation, we chose the most significant differently expressed miRNA‐miR‐27‐3p. Our data showed that miRNA‐27 was significantly higher in exosomes of non‐OHSS patients compared with supernatant of follicular fluid, which suggested that miR‐27 might exercise its function through exosomes in follicular fluid. The miR‐27‐enriched exosomes might be secreted by granulosa cells, follicular membrane cells and other cell types during OHSS process, affect crosstalk between granulosa cells and follicle environment. To investigate how miR‐27 causes granulosa cell apoptosis, we searched 4 miRNA databases to identify potential target genes and then narrowed on SPRY2. Besides being a member of the Sprouty family and the activity of receptor tyrosine kinase signalling, SPRY2 is required for growth factor‐stimulated translocation to membrane ruffles.^36^ Additionally, it has been reported that SPRY2 was significantly high in granulosa cells of OHSS patients and regulates the expression of COX2/PGE2.^37^ Our findings also demonstrated that miR‐27 negatively regulates SPRY2 mRNA and protein levels. Moreover, we used the 3’UTR luciferase analysis to show that miR‐27 directly targets the 3’UTR of SPRY2. Our knockdown studies with SPRY2 siRNA demonstrated that SPRY2 mediates the effects of miR‐27 on ERK signalling. Recent studies have reported an association between SPRY2 and miR‐27‐3p.^36^ SPRY2 also regulates EGFR trafficking and cell signalling, SPRY2 knockdown cells significantly promoted EGFR endocytosis and significantly reduced extracellular signal‐regulated kinase (ERK) phosphorylation and EGFR expression. The expression of EGFR in OHSS patients was up‐regulated, causing an up‐regulation of VEGF expression.^19^ Because of many existing reports, this work did not put much emphasis on the effect of ERK on VEGF. Nrf2 is an important transcription factor that influences the expression of antioxidant defence genes. This is because ERK usually activates the downstream Nrf2 pathway, thus activating the downstream antioxidant stress pathway.^38^ Whether miR‐27 could regulate Nrf2 and its downstream proteins, we used transfection of miR‐27 mimic and miR‐27 inhibitor. Due to the inhibitory effect of the high concentration of miR‐27 on SPRY2, it can regulate the anti‐oxidative stress of granulosa cells. In addition, MiR‐27 induce apoptosis of granulosa cells, which reduces the secretion of VEGF and other factors, and thus improve the symptoms of OHSS.

To our knowledge, the present investigation is a pioneer study demonstrating that the exosomal miRNAs participate in the development of OHSS. Exosomes might be one of the mechanism of granulosa cells apoptosis regulation in OHSS. Future studies about specific exosomal or non‐exosomal miRNAs and their roles in OHSS are needed to further investigate. In conclusions, we found different profile of miRNAs derived from follicular fluid in OHSS patients and normal control. We found miR‐27 in exosomes could inhibit ERK/Nrf2 signalling pathways by targeting SPRTY2 in granulosa cells, increases ROS levels and cells apoptosis. Our findings suggest the importance of exosomes as extracellular mediators in the pathophysiology of OHSS. One limitation of this study is the lack of animal model validation we might be able to unravel the relevant mechanisms in OHSS animal models in the future work.

## CONFLICT OF INTEREST

The authors confirm that there are no conflicts of interest.

## AUTHOR CONTRIBUTIONS

**Kailu Liu:** Data curation (lead); Formal analysis (lead); Investigation (lead); Project administration (lead); Writing‐original draft (lead). **Weijie Yang:** Data curation (equal); Project administration (lead). **Mengting Hu:** Data curation (supporting); Formal analysis (supporting). **Wenxiu Xie:** Formal analysis (equal); Resources (equal). **Jingyu Huang:** Data curation (lead); Methodology (equal); Project administration (equal). **Meiting Cui:** Formal analysis (equal); Methodology (equal). **Xi He:** Writing‐original draft (lead); Writing‐review & editing (lead). **Xiaowei Nie:** Data curation (equal); Funding acquisition (lead); Investigation (lead); Methodology (lead); Project administration (lead).

## Supporting information

Fig S1

## Data Availability

The data that support the findings of this study are available from the corresponding author upon reasonable request.
