# Supplementary figures and images for: Exosomal miR‐27 negatively regulates ROS production and promotes granulosa cells apoptosis by targeting SPRY2 in OHSS
Source: J Cell Mol Med. 2021 Feb 27;25(8):3976–90. doi: 10.1111/jcmm.16355 (PMC8051746; doi:10.1111/jcmm.16355)

Figure.1

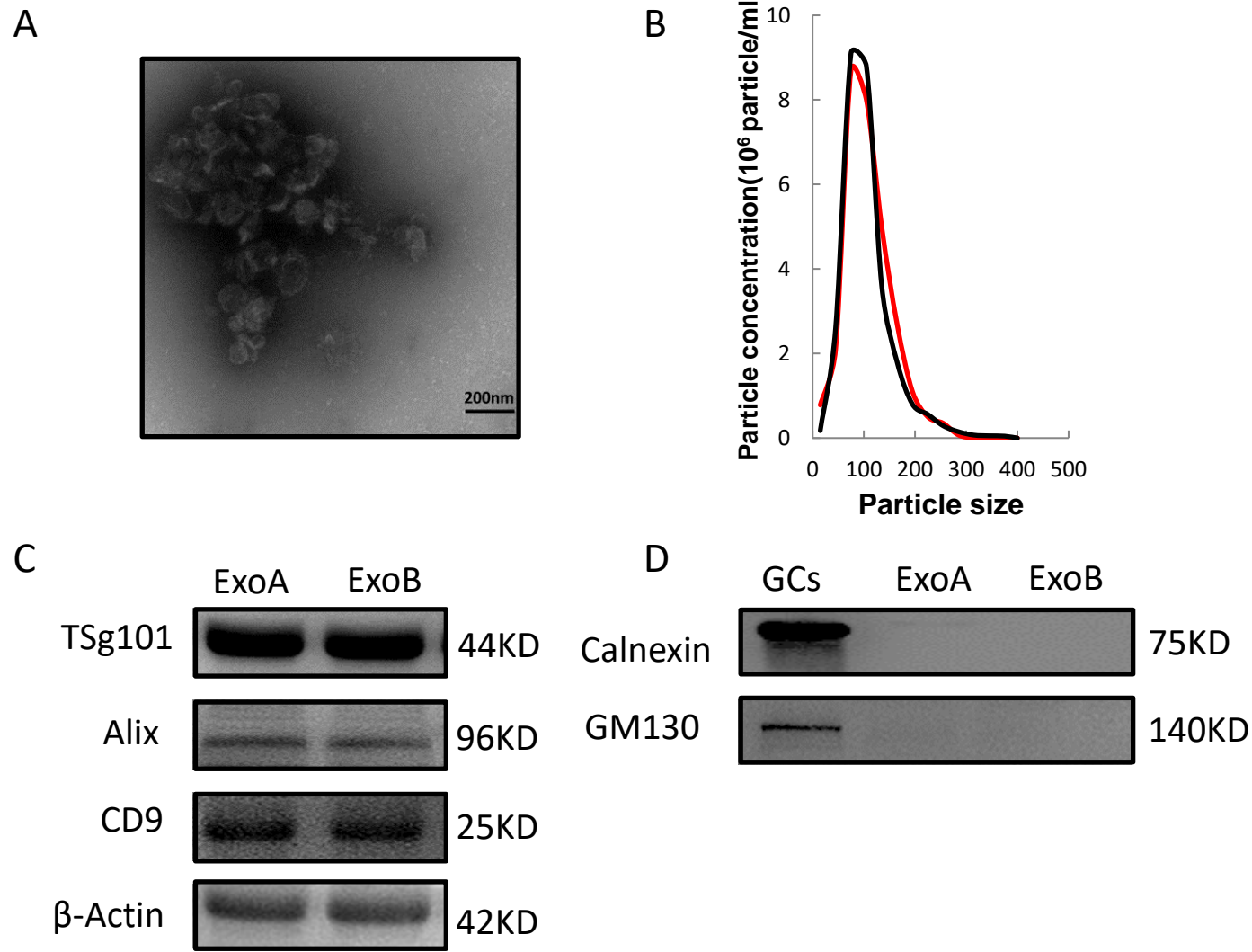

Figure.2

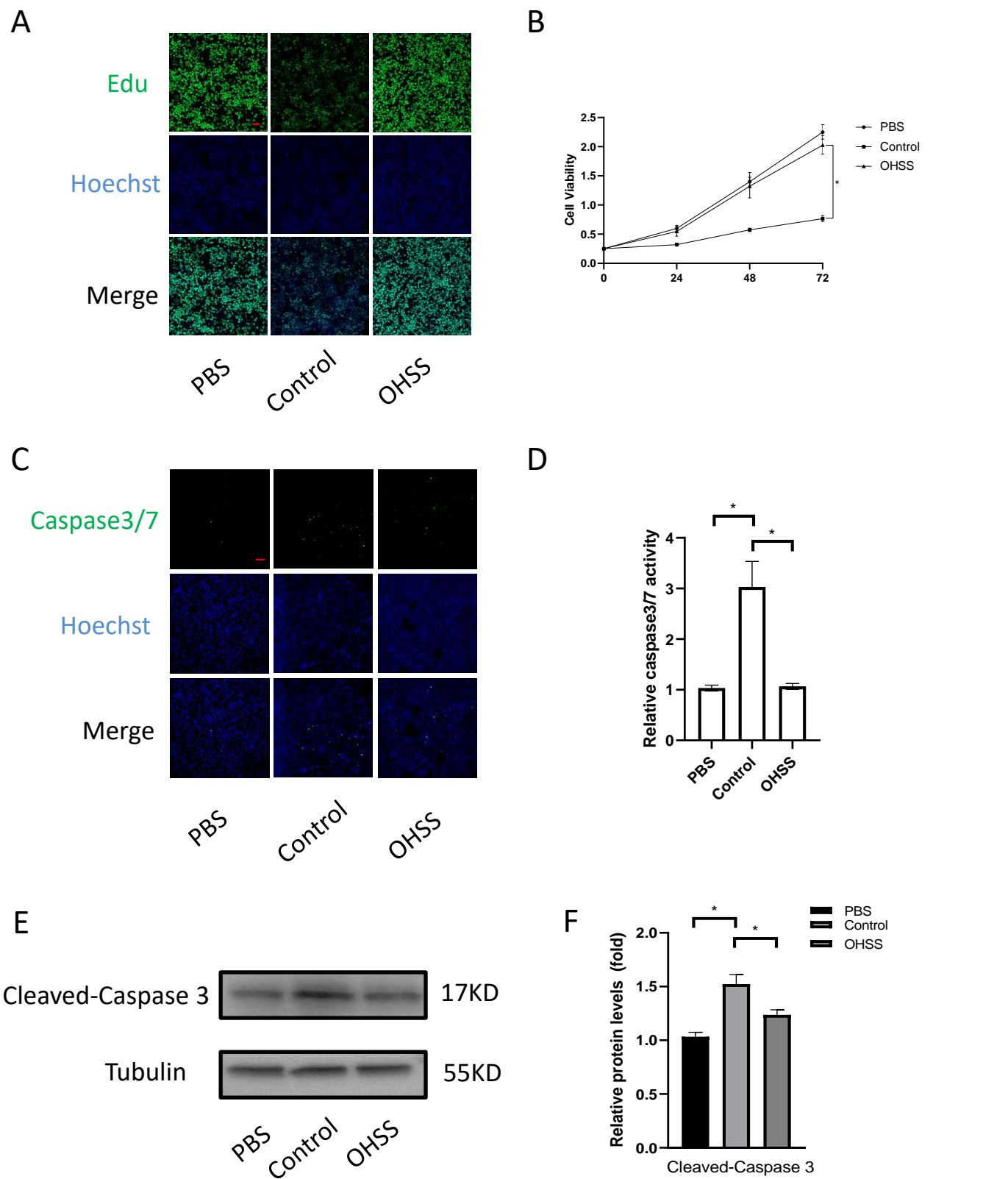

Figure.3

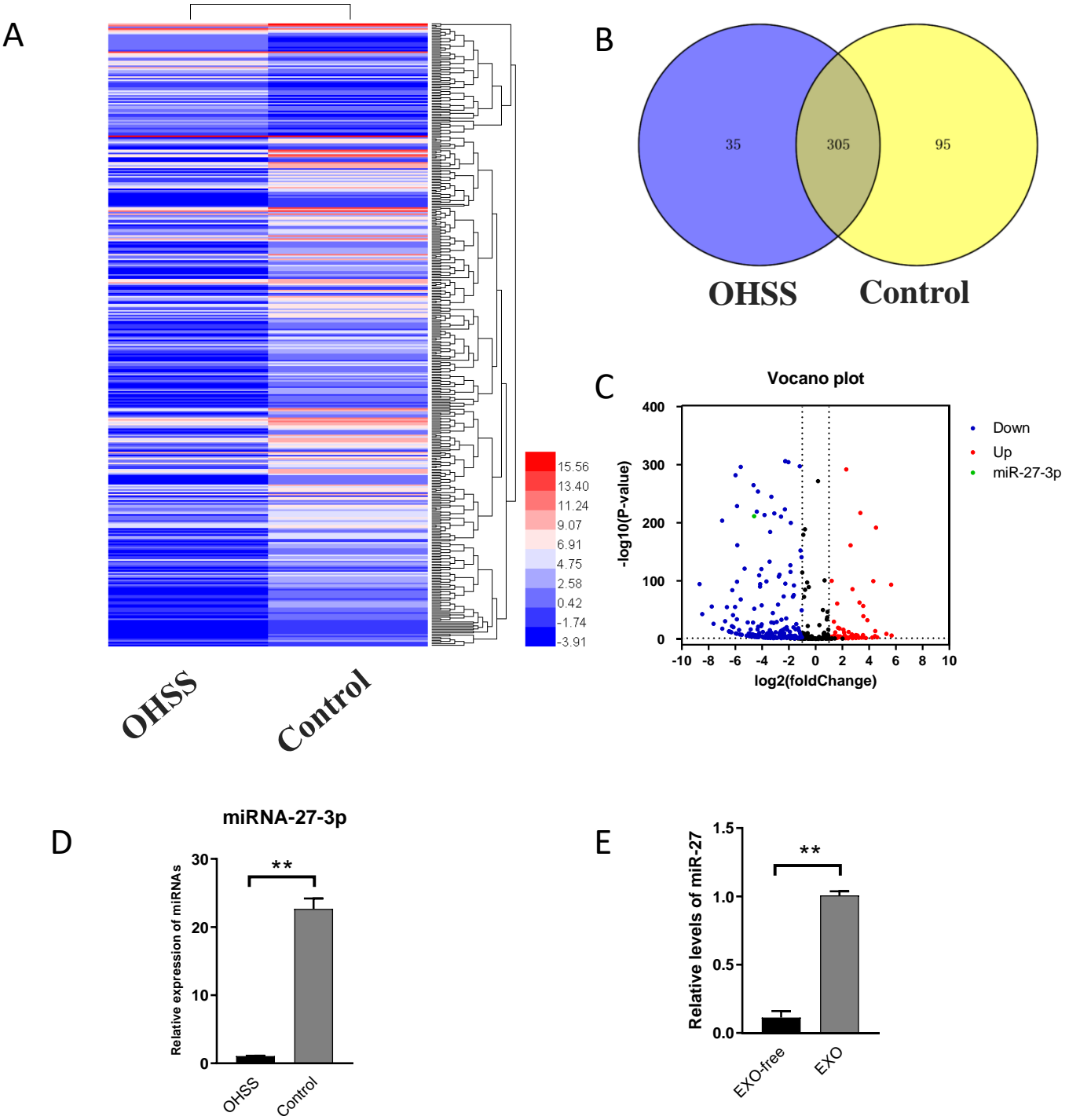

Figure.4

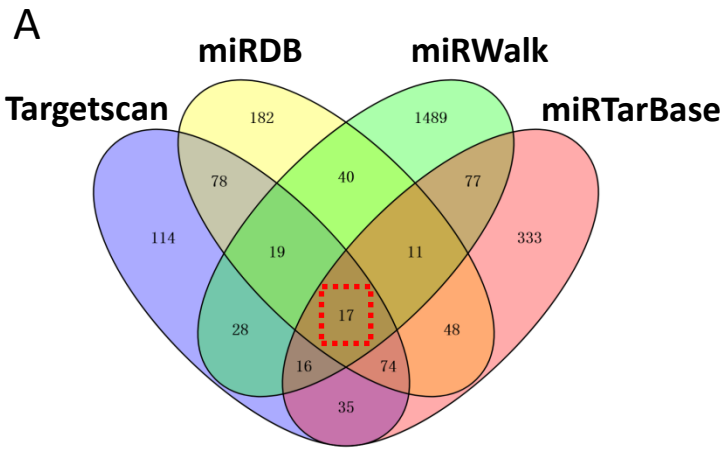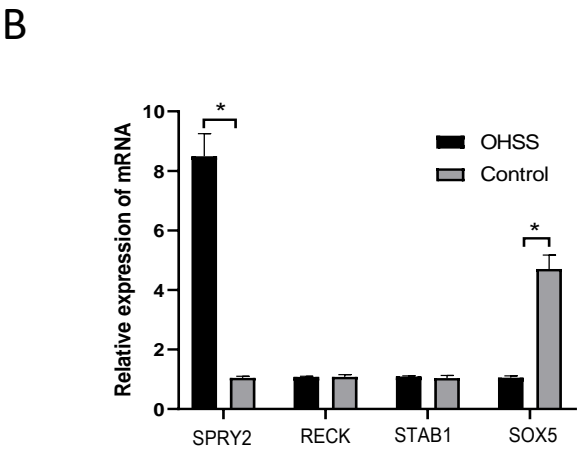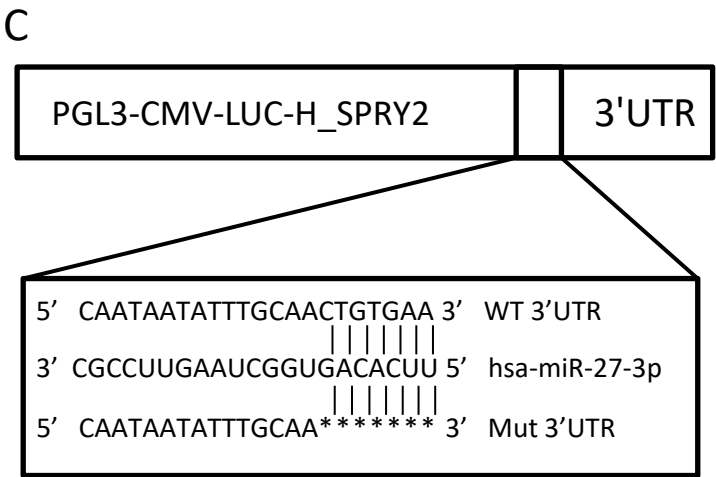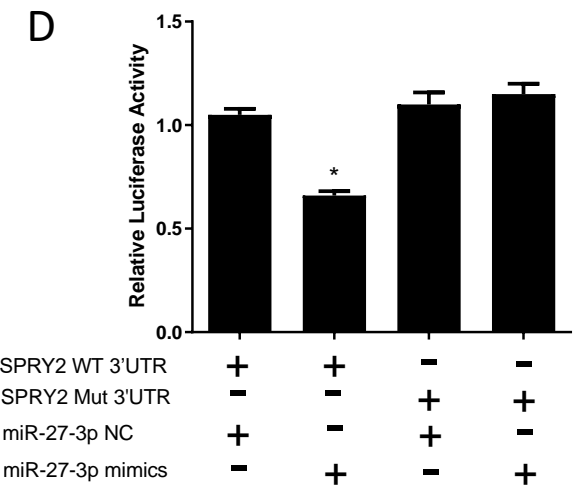

Figure.5

A

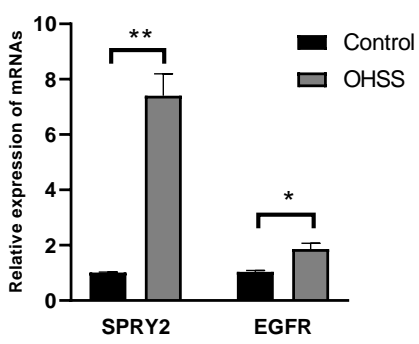

B

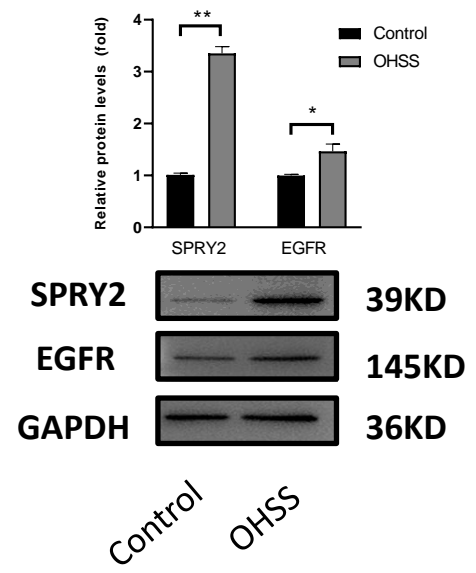

C

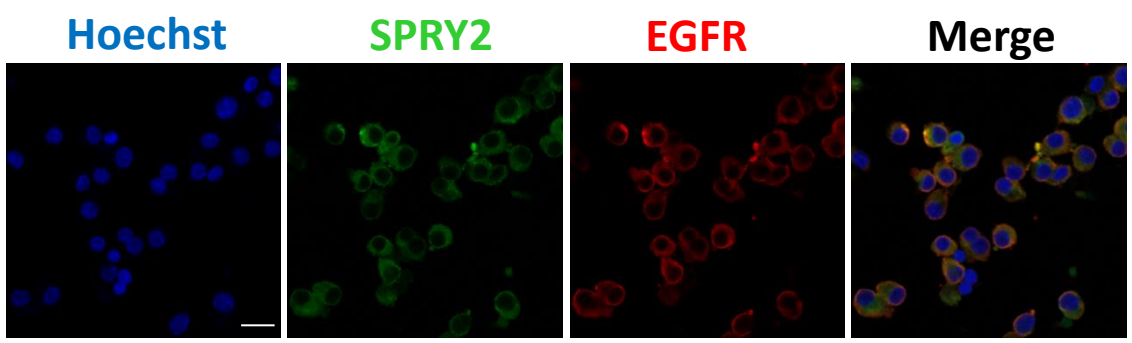

D

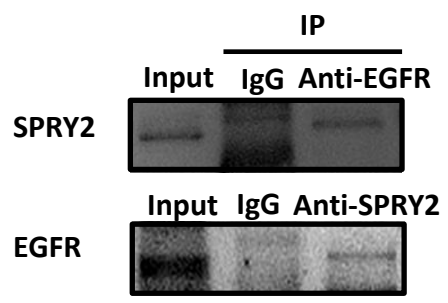

E

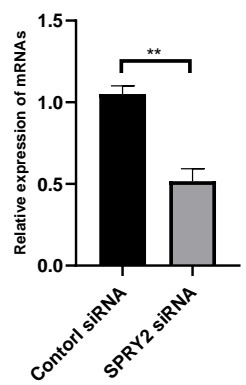

F

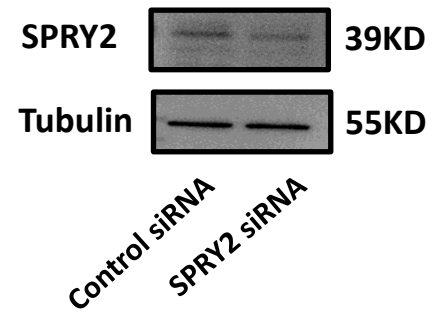

G

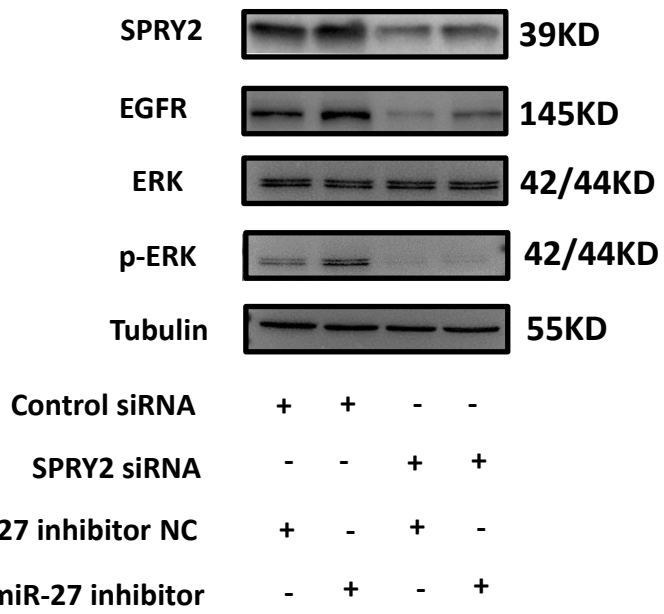

H

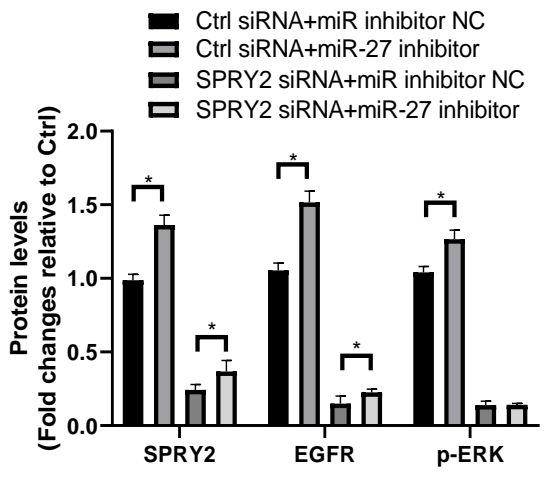

Figure.6

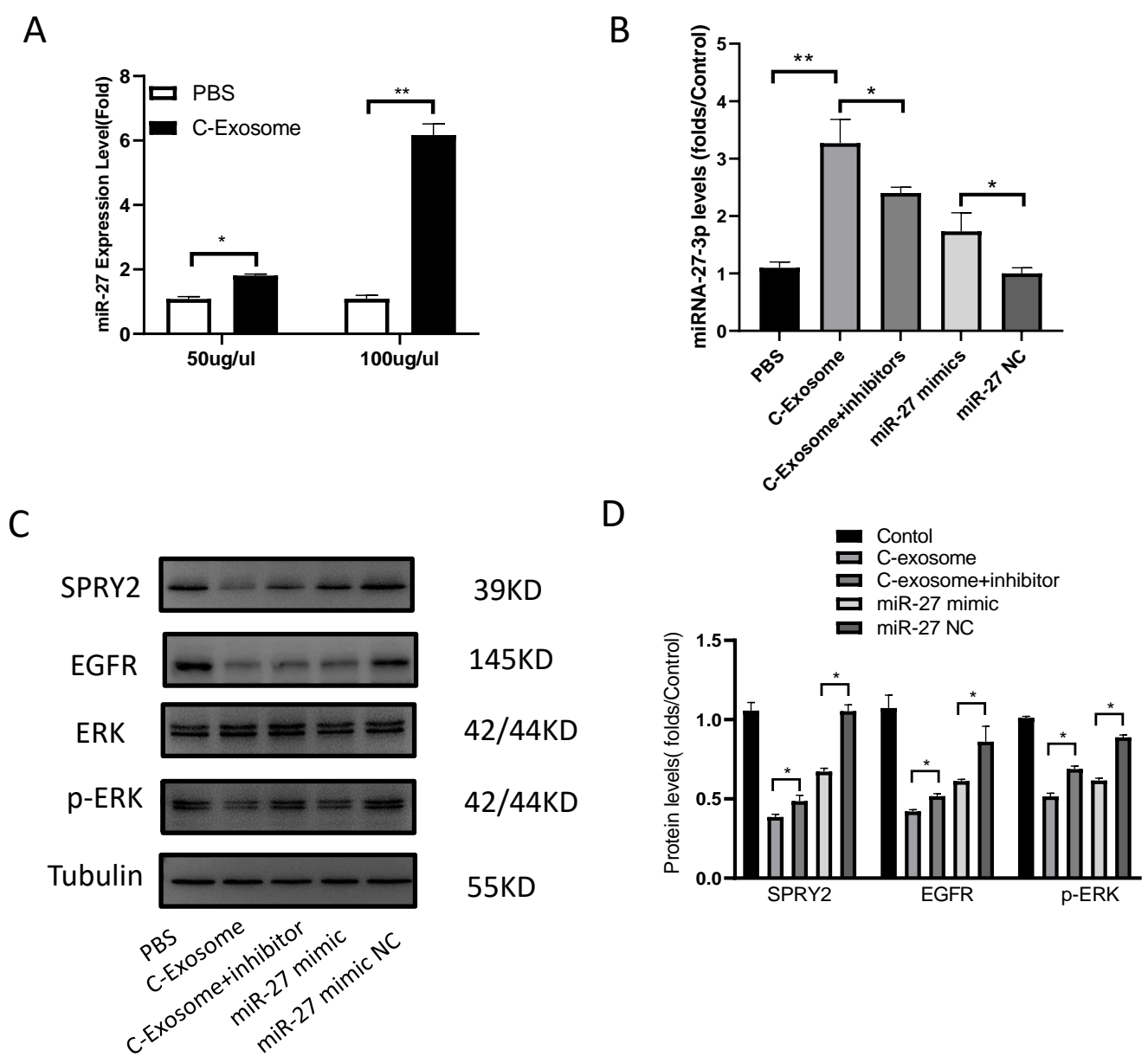

Figure.7

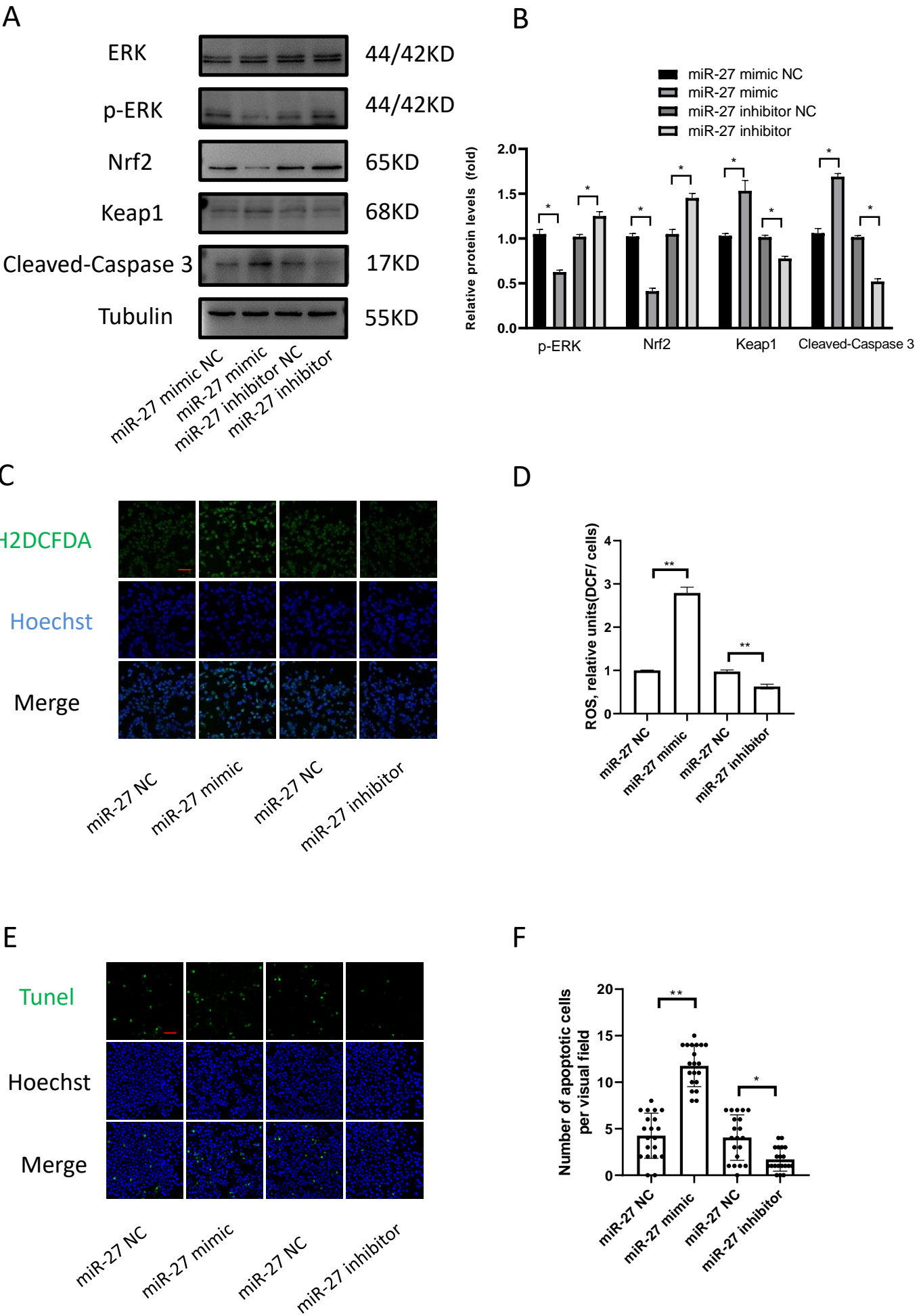

Supplyment Figure.1

A

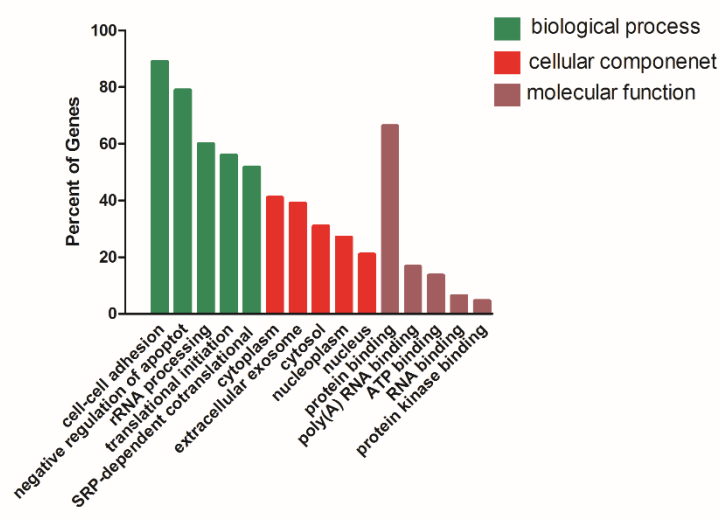

B

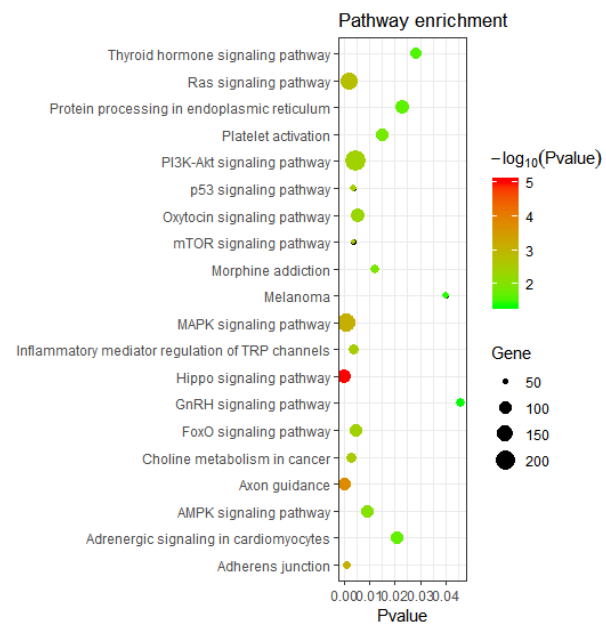

C

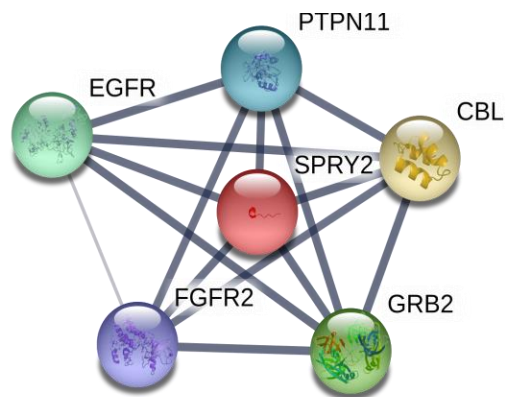

Supplement: Supplementary file 1 — Fig S1 [file JCMM-25-3976-s001.pdf]
